# MSI1 Promotes the Expression of the GBM Stem Cell Marker CD44 by Impairing miRNA-Dependent Degradation

**DOI:** 10.3390/cancers12123654

**Published:** 2020-12-05

**Authors:** Rebecca Pötschke, Jacob Haase, Markus Glaß, Sebastian Simmermacher, Claudia Misiak, Luiz O. F. Penalva, Caspar D. Kühnöl, Stefan Hüttelmaier

**Affiliations:** 1 Charles Tanford Protein Center, Institute of Molecular Medicine, Sect. Molecular Cell Biology, Martin Luther University Halle-Wittenberg, 06120 Halle, Germany; rebeccapoetschke@googlemail.com (R.P.); jacob.haase@medizin.uni-halle.de (J.H.); markus.glass@medizin.uni-halle.de (M.G.); claudia.misiak@medizin.uni-halle.de (C.M.); 2 Department of Pediatric Hematology/Oncology, University Clinics Halle, 06120 Halle, Germany; 3 Department of Neurosurgery, University Clinics Halle, 06120 Halle, Germany; sebastian.simmermacher@uk-halle.de; 4 Department of Cell Systems and Anatomy, University of Texas Health Science Center at San Antonio, San Antonio, TX 78229, USA; penalva@uthscsa.edu; 5 Greehey Children’s Research Institute, University of Texas Health Science Center at San Antonio, San Antonio, TX 78229, USA

**Keywords:** MSI1, Musashi1, CD44, cancer stem cell, glioblastoma, GBM, recurrence, miRNA, luteolin

## Abstract

**Simple Summary:**

Glioblastoma (GBM) is the most lethal brain tumor with a median survival rate of approximately 14 months. GBM patients commonly suffer from tumor recurrence, indicating that populations of chemo/radio-resistant stem cell-like tumor cells survive treatments. Here we reveal that the neuronal stem cell marker Musashi1 (MSI1) is highly expressed in primary GBM and recurrences. We identify a novel regulatory role of MSI1 in GBM-derived cell lines and patient-derived tumorspheres, the enhancement of stemness marker expression, here demonstrated for CD44. Furthermore, we provide a rationale for MSI1-centered therapeutic targeting strategies to improve treatment options of this chemo/radio-resistant malignancy.

**Abstract:**

The stem cell marker Musashi1 (MSI1) is highly expressed during neurogenesis and in glioblastoma (GBM). MSI1 promotes self-renewal and impairs differentiation in cancer and non-malignant progenitor cells. However, a comprehensive understanding of its role in promoting GBM-driving networks remains to be deciphered. We demonstrate that MSI1 is highly expressed in GBM recurrences, an oncologist’s major defiance. For the first time, we provide evidence that MSI1 promotes the expression of stem cell markers like CD44, co-expressed with MSI1 within recurrence-promoting cells at the migrating front of primary GBM samples. With GBM cell models of pediatric and adult origin, including isolated primary tumorspheres, we show that MSI1 promotes stem cell-like characteristics. Importantly, it impairs CD44 downregulation in a 3′UTR- and miRNA-dependent manner by controlling mRNA turnover. This regulation is disturbed by the previously reported MSI1 inhibitor luteolin, providing further evidence for a therapeutic target potential of MSI1 in GBM treatment.

## 1. Introduction

Glioblastoma (GBM) is the most prevalent and lethal primary brain tumor (WHO grade IV glioma), with a median survival rate of approximately 14 months. Unlike other solid tumors, GBM show aggressive invasion into surrounding tissues, but rarely metastasize to distant organs [1]. GBM patients commonly suffer from tumor recurrence, indicating that populations of radio- and chemoresistant tumor cells survive treatments. These cells are likely GBM cancer stem cells (GBM-CSCs) [2]. By investigating single-cell RNA sequencing data (scRNA-seq), besides neurodevelopmental lineages, the existence of GBM-CSCs have been validated most recently [3]. These cells usually are identified by expressing SOX2, NANOG, OLIG2, MYC, NES, Musashi1 (MSI1) and the surface markers CD133 (PROM1), CD44, ITGA6 or L1CAM [1]. Although research shed light on the biology and occurrence of GBM by using leading-edge transcriptomic and (epi-) genomic profiling techniques, no significant advancement on patient survival could be achieved over the past decades. Thus, the elucidation of key mechanisms remains crucial in order to identify effective GBM targeting strategies.

The RNA-binding protein (RBP) MSI1 functions as a regulator of differentiation by maintaining pluripotency and self-renewal of neural stem and progenitor cells [4,5]. It is re-expressed in various malignancies, including glioma, colorectal, mammary and lung cancer, suggesting an oncofetal pattern of expression [6,7,8,9]. Interestingly, MSI1 is claimed to be a marker for GBM-CSCs [10]. In previous studies, the main role of MSI1 was reported to rely on the 3′UTR (3’ untranslated region)-dependent regulation of mRNA translation, as demonstrated for NUMB [4]. In GBM and other cancer models, MSI1 was reported to promote a stem-like cell state by modulating self-renewal, migration/invasion, differentiation, chemoresistance and recurrence [11,12,13,14,15,16].

In this study, we report on a novel mechanism for the RBP MSI1—the 3′UTR- and microRNA (miRNA)-dependent downregulation of CD44 mRNA expression in GBM, which is inhibited by luteolin, previously reported to impair the MSI1–RNA association in GBM-derived cells [17]. By evaluating scRNA-seq data, we show that MSI1 and CD44 mRNAs are co-expressed at the invasive front of patient-derived GBM samples in single neoplastic cell populations [18]. Our studies indicate that MSI1 is a potent and druggable enhancer of CSC marker expression and properties in GBM, strongly emphasizing to expedite clinical evaluation and improvement of MSI1-directed inhibitors for cancer treatment.

## 2. Results

### 2.1. MSI1 Conveys a Pro-Oncogenic Role in GBM Cells In Vitro

**Figure 1:**
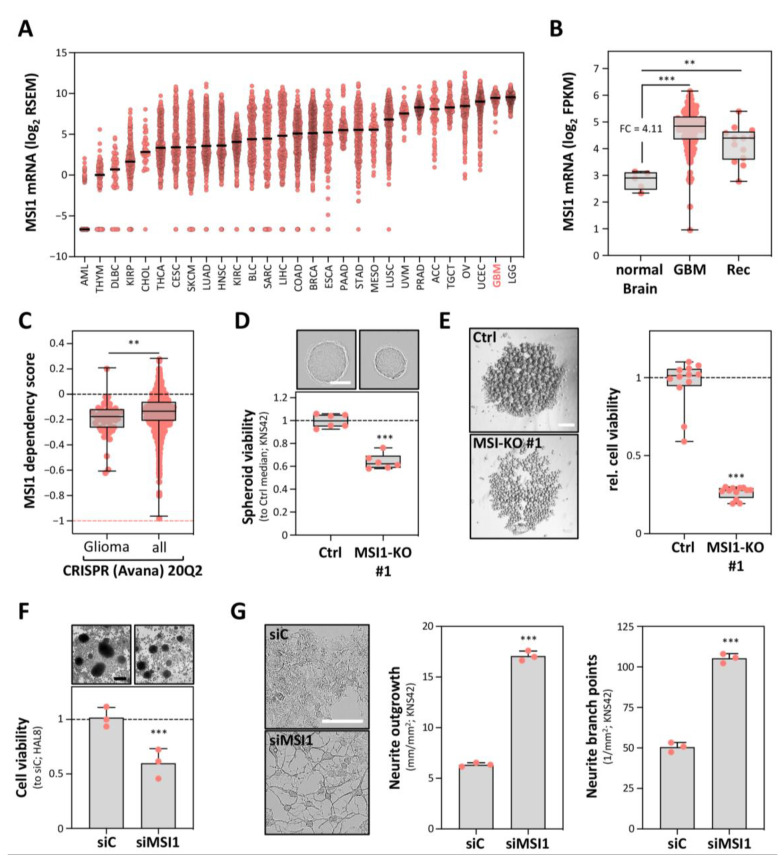
Musashi1 (MSI1) conveys a pro-oncogenic role in glioblastoma (GBM)-derived cells. (**A**) Scatter plot presentation of MSI1 mRNA expression in all TCGA cohorts, provided by cBioPortal. (**B**) Box plot presentation of MSI1 mRNA expression for primary GBM and paired recurrence (Rec) samples, compared to normal brain tissue, derived from TCGA. (**C**) Box plot presentation of MSI1 dependency scores, determined for glioma-derived cell lines and for all provided cell lines from the DepMap database (Avana 20Q2). Dashed line (salmon) indicates the essentiality threshold. (**D**) Spheroid viability of the Cas9-only transfected (Ctrl) and MSI1 knockout cell clones (*n* = 6 per condition) as in (Figure S1B,C), determined by CellTiter-GLO, 72 h after seeding. Upper panel, representative images of the spheroids; scale bar, 150 µm. Lower panel: box plots showing the spheroid viability normalized to the median viability of the Cas9-only transfected cell clone (set to one). (**E**) Anoikis resistance of the Cas9-only transfected (Ctrl) and MSI1 knockout cell clones (*n* = 12 per condition) 7 days after seeding. Left panel, representative images of the cell aggregates; scale bar, 300 µm. Right panel, box plots showing the cell viability as determined in (D). (**F**) Relative cell viability for the siC- and siMSI1-transfected primary tumorspheres (HAL8), determined by CellTiter-GLO, 72 h post-transfection. Upper panel, representative images of the primary tumorspheres; scale bar, 150 µm. Lower panel, box plots showing the relative cell viability, normalized to the median of the siC-transfected cells (set to one). (**G**) Neurite growth parameters for the siC- and siMSI1-transfected KNS42 cells, 72 h post-transfection. Left panel: representative images of the KNS42 cells. Middle and right panels, quantification of the neurite outgrowth and branch points. Error bars indicate the standard deviation from at least three independent experiments. Statistical significance was determined by Mann–Whitney tests (**B**,**C**) and Student’s *t*-tests (**D**–**G**) (***, *p* ≤ 0.001; **, *p* ≤ 0.01).

MSI1 is exceptionally highly expressed in gliomas, including GBM (Figure 1A and Figure S1A) [19,20]. In agreement with the proposed role of MSI1 in glioma-initiating stem or progenitor cells, its mRNA expression was also found comparably high in tumor recurrences (Figure 1B) [19,21]. CRISPR/Cas9-determined essentiality scores support a pivotal role of MSI1 in glioma-derived cells, since in median, these depend on MSI1 significantly more than other cancer cells (Figure 1C) [22]. Notably, however, these analyses suggest that, at least in vitro, MSI1 is not core essential (dependency score < −1).

To evaluate the role of MSI1 in GBM cell models, we generated CRISPR/Cas9-mediated *MSI1* gene knockouts in the pediatric GBM-derived cell line KNS42 (Figure S1B). Consistent with findings in other cell models, MSI1 deletion significantly impaired the proliferation and vitality of both adherently growing cells as well as in 3D tumor spheroid models (Figure 1D and Figure S1C,D). This proliferation/vitality-promoting role was substantially more prominent when investigating tumor cell fate under low adhesion and mitogen deprivation. In such anoikis-resistance analyses, MSI1 deletion severely impaired tumor cell vitality (Figure 1E). In support of deletion studies in GBM-derived cell lines, MSI1 depletion by an siRNA pool impaired the viability of the patient-derived (patient age: 76 years) GBM tumorspheres, termed HAL8 (Figure 1F and Figure S1G,H). In these, MSI1 showed strong co-expression with a variety of GBM stem cell markers, as revealed by RNA-seq and flow cytometry (see Figure S1E,F; Table S1).

To test a potential role of MSI1 in neural differentiation, we monitored how MSI1 depletion affects cell morphology and the expression of the astrocyte differentiation marker GFAP (glial fibrillary acidic protein). In KNS42 cells, MSI1 knockdown substantially increased neurite outgrowth and branching (Figure 1G and Figure S1G,H). Associated with this morphologically neural-like re-differentiation, GFAP expression was significantly enhanced in both KNS42 as well as primary HAL8 tumorspheres (Figure S1G). These findings are in accord with the previously observed low expression of GFAP in MSI1-positive tumor cells, which are typically surrounded by MSI1-negative cells with high GFAP expression [8]. Collectively, these studies indicated that MSI1 promotes a de-differentiated, stem-like tumor cell phenotype in distinct glioma cell models of pediatric as well as adult origin.

### 2.2. MSI1 Promotes CD44 Expression in GBM Cells

**Figure 2:**
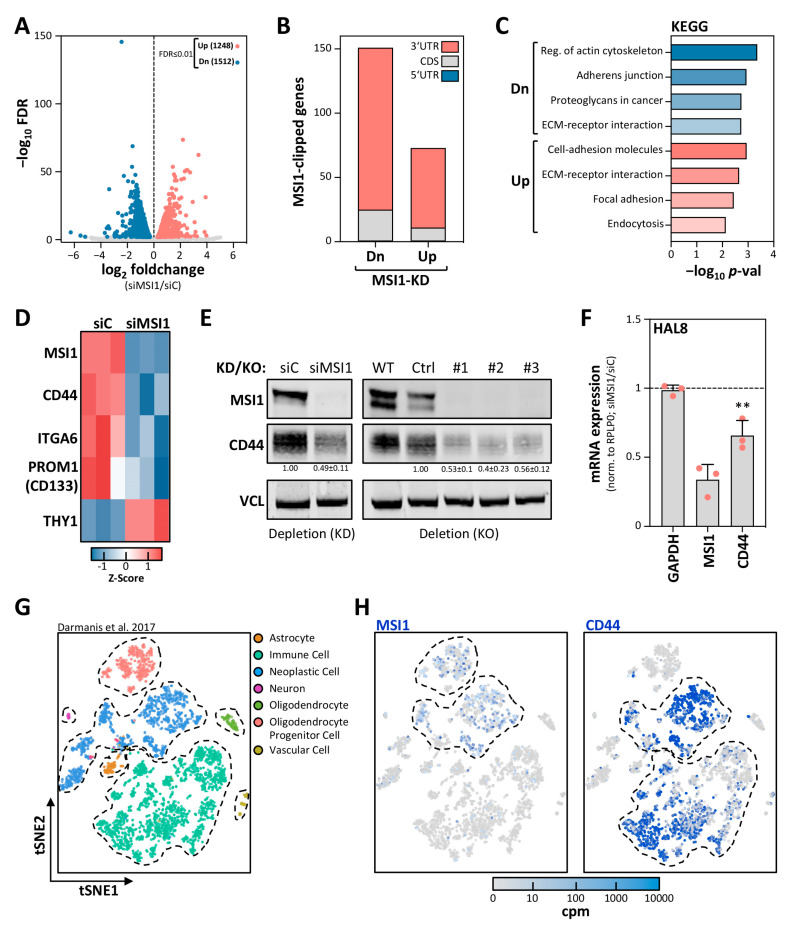
MSI1 promotes CD44 expression in GBM-derived cells. (**A**) Volcano plot of the mRNA fold changes plotted against the FDR for siMSI1 versus siC in KNS42 cells, determined by mRNA-seq. Significantly differentially expressed genes (FDR ≤ 0.01) are highlighted in blue (downregulated) and red (upregulated). Numbers of differentially expressed genes are indicated. (**B**) The association of MSI1 protein with mRNA cis-elements, determined by CLIP [12], of significantly down- or upregulated genes (FDR ≤ 0.01) upon MSI1 depletion as in (**A**) is depicted. (**C**) KEGG analyses performed on all genes identified to be clipped in (**B**), by using Enrichr [25]. (**D**) Heatmap presentation depicting the expression of significantly differentially expressed (FDR ≤ 0.01) CSC marker genes (as in Figure S1E) in KNS42 cells, generated with Heatmapper [29]. (**E**) Representative Western blot analysis of indicated proteins of siC- and siMSI1-transfected KNS42 cells. VCL served as the negative and loading control. (**F**) Relative mRNA expression determined by RT-qPCR for the indicated transcripts upon transient MSI1 depletion in the primary tumorspheres HAL8, 72 h post-transfection. (**G**,**H**) 2D-tSNE analysis presentation of all single cells included in the study (*n* = 3589) by Darmanis et al. (2017) [3,28]. Distinct identified types of cell clusters are indicated (**G**). MSI1 and CD44 expression levels are indicated for each single cell in increasing shades of blue, as indicated by a color code (**H**). Error bars indicate the standard deviation from three independent experiments. Statistical significance was determined by Student’s *t*-test (**, *p* ≤ 0.01).

MSI1 has been reported to control mRNA translation [21] and was also suggested to modulate target transcript turnover [11,23], although a role in mRNA decay has never been tested thoroughly. Our studies, however, suggested that the stabilization of mRNAs by MSI1 substantially contributes to the enhancement of a de-differentiated, stem-like glioma cell phenotype by enhancing the expression of the stem cell markers. This was addressed by mRNA-sequencing in KNS42 cells depleted for MSI1 (Figure 2A). The knockdown of MSI1 led to a substantial deregulation of mRNA abundance. However, among the transcripts significantly decreased, we observed a striking enrichment of the transcripts identified by CLIP to associate with MSI1 in U251MG cells [12] (Figure 2B). Intriguingly, among the deregulated transcripts with reported MSI1 association, the prime cis-elements recognized by MSI1 were 3′UTRs, as recently reported [12,24]. KEGG pathway analyses [25] revealed an enrichment of candidate target transcripts in adhesion and ECM interaction/regulation pathways (Figure 2C), supporting a role of MSI1 in tumor cell adhesion, migration and invasion [12].

To further shed light on the impact of MSI1 on glioma cell differentiation and stem cell characteristics, we focused on the expression of reported GBM-CSC markers [1,26,27], co-expressed with MSI1 in HAL8 tumorspheres (see Supplementary Figure S1E,F). Intriguingly, we observed significant (FDR ≤ 0.01) downregulation of CD44, CD133, ITGA6 and THY1 (Figure 2D, Table S2). Out of these, a consistent MSI1–mRNA association was reported for CD44 in U251MG cells by CLIP [12]. This suggested the CD44 mRNA as a novel candidate target mRNA of MSI1, potentially stabilized by the protein in GBM-derived cells. This was further analyzed by monitoring CD44 expression in KNS42 and HAL8 cells upon MSI1 knockdown (KD) and deletion (KO). In KNS42 cells, the depletion and deletion of MSI1 led to substantial downregulation of CD44 protein (Figure 2E). Likewise, we observed that MSI1 depletion in HAL8 cells, where deletion proved nonviable, resulted in downregulated CD44 mRNA levels (Figure 2F).

Recently, scRNA-seq revealed a distinct CD44-positive lineage among GBM cells within the migrating front of GBM cells [3,28]. Investigation of scRNA-seq data of these studies, deposited in the single-cell expression atlas, revealed substantial co-expression of MSI1 and CD44 only in neoplastic cells (Figure 2G,H and Figure S1I). These findings linked the regulation of CD44 expression by MSI1 to the first description of their co-occurrence in the same neoplastic cell population observed at the invasive front of patient-derived GBM.

### 2.3. The MSI1 Inhibitor Luteolin Impairs CD44 Expression in GBM

**Figure 3:**
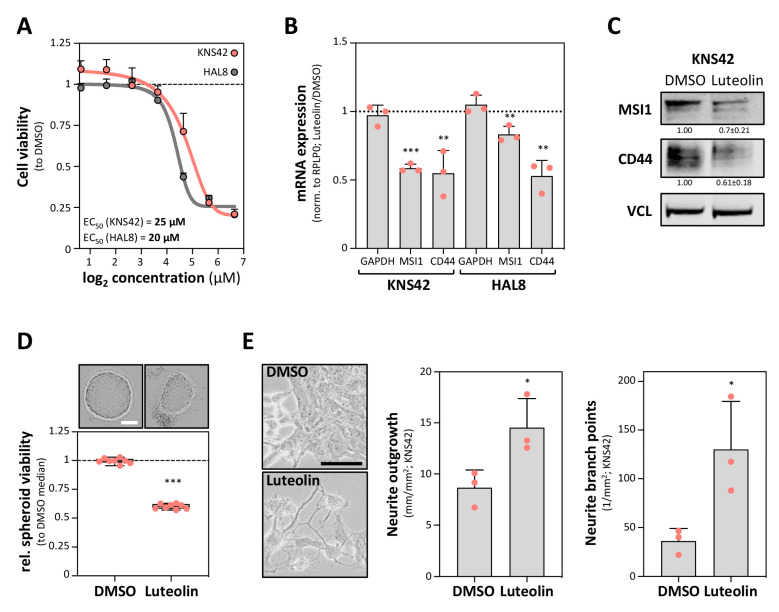
A luteolin treatment impairs CD44 expression and induces differentiation. (**A**) Cell viability ratios (to DMSO) of KNS42 cells and primary tumorspheres HAL8 exposed to different concentrations of MSI1-targeting luteolin for 72 h. Response curves and EC_50_ values are shown. (**B**) Relative mRNA expression determined by RT-qPCR for the indicated transcripts upon luteolin treatment at EC_50_ concentrations to DMSO after 72 h in KNS42 cells and the primary tumorspheres HAL8. (**C**) Representative Western blot analyses of the indicated proteins in KNS42 cells exposed to luteolin at EC_50_ concentration and DMSO. VCL served as the negative and loading control. (**D**) Spheroid viability of the KNS42 cells exposed to luteolin at EC_50_ concentration and DMSO, determined by CellTiter-GLO, 72 h after seeding. Upper panel, representative images of the spheroids; scale bar, 150 µm. Lower panel: box plots showing the spheroid viability normalized to the median viability of the DMSO-treated cells (set to one). (**E**) Neurite growth parameters for the KNS42 cells exposed to luteolin at EC_50_ concentration and DMSO after 72 h. Left panel: representative images of the KNS42 cells. Middle and right panels, quantification of the neurite outgrowth and branch points. Error bars indicate the standard deviation from three independent experiments. Statistical significance was determined by Student’s *t*-test (***, *p* ≤ 0.001; **, *p* ≤ 0.01; *, *p* ≤ 0.05).

Previous studies reported that the MSI1–RNA association and MSI1-driven enhancement of pro-oncogenic target genes in GBM-derived cells is impaired by luteolin [17]. In agreement, luteolin impaired the vitality of KNS42 cells as well as primary HAL8 tumorspheres, with half-maximal effective concentrations (EC_50_) of 25 µM and 20 µM, respectively (Figure 3A). Intriguingly, CD44 expression was reduced by luteolin treatment in both KNS42 cells as well as HAL8 tumorspheres (Figure 3B,C). Whereas MSI1 mRNA was barely affected in HLA8 tumorspheres, MSI1 mRNA and protein abundance were more prominently reduced in KNS42 cells. We therefore tested if MSI1 downregulation could result from enhanced differentiation induced by luteolin treatment. In support of this notion, KNS42 cells treated with luteolin at EC_50_ concentrations for 72 h showed significantly reduced spheroid viability, increased neurite outgrowth and elevated neurite branching (Figure 3D,E). Thus, luteolin treatment was associated with reduced CD44 expression and elevated neural-like differentiation, as observed upon MSI1 depletion in GBM-derived cell models. This supported the MSI1-dependent regulation of CD44 expression and the suitability of luteolin-directed inhibition of MSI1 as a lead therapeutic concept.

### 2.4. MSI1 Promotes CD44 Expression in a 3′UTR Dependent Manner

**Figure 4:**
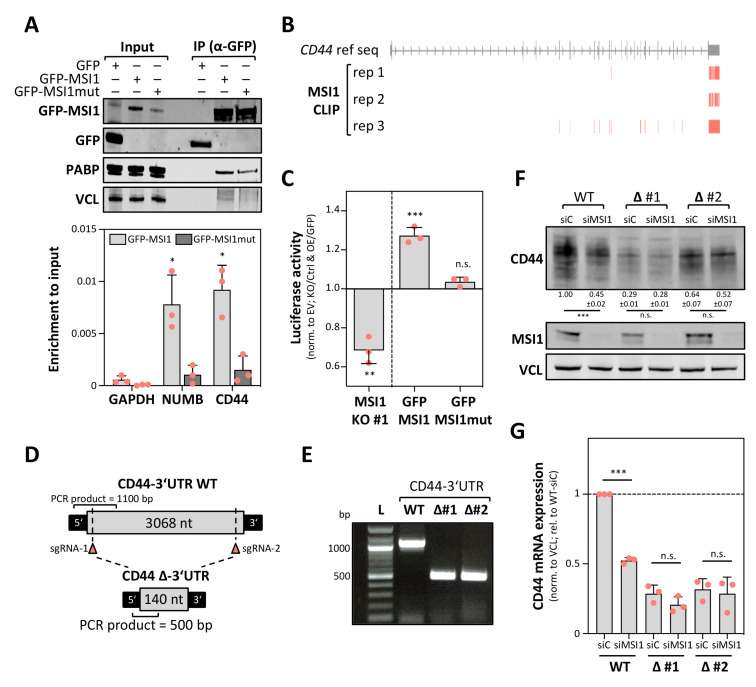
MSI1 promotes CD44 expression in a 3′UTR-dependent manner. (**A**) Representative Western blot analysis (upper panel) of the indicated proteins and RT-qPCR analyses (lower panel) of mRNAs co-purified with GFP, GFP-MSI1 or RNA-binding-deficient GFP-MSI1mut by immunoprecipitation. The enrichment of the indicated mRNAs is shown upon input normalization. VCL and GAPDH served as the loading and negative controls. (**B**) MSI1 CLIP-derived peak coordinates [12], mapped to the genomic CD44 reference sequence, are depicted. (**C**) Luciferase activity of the reporters comprising the full-length CD44-3′UTR determined in MSI1-deleted (MSI1-KO #1) KNS42 cell clones (relative to Cas9-only transfected) and KNS42 cells expressing GFP-MSI1 or GFP-MSI1mut (relative to GFP). A reporter comprising the vector-encoded 3′UTR (EV) served as the control, as shown in (S2B). (**D**) Schematic depicting the generation of a CD44-3′UTR deletion. Small guide RNA (sgRNA) sites for specific cleavage are indicated upstream of the stop codon and downstream of the last polyadenylation signal. (**E**) Representative genomic DNA analysis of the Cas9-only transfected (WT) and CD44-3′UTR deleted (∆) KNS42 cell clones. Respective PCR product sizes are shown, as indicated in (D). (**F**,**G**) Representative Western blot analysis of the indicated proteins (**F**) and RT-qPCR analysis of indicated transcripts (**G**) in CD44-3‘UTR WT (Cas9-transfected only) and two individual CD44-∆3‘UTR (biallelic CD44-3′UTR deletion) KNS42 cell clones, 72 h upon siC and siMSI1 transfection. Error bars indicate the standard deviation from three independent experiments. Statistical significance was determined by Student’s *t*-test (***, *p* ≤ 0.001; **, *p* ≤ 0.01; *, *p* ≤ 0.05; n.s., not significant).

Previous CLIP analyses used for the identification of candidate MSI1 target mRNAs in this study were performed in U251MG cells [12]. Thus, we investigated if MSI1 associates with the CD44 mRNA also in KNS42 cells using RNA co-immunoprecipitation (RIP). To this end, the RNA and protein association of GFP-MSI1 and an RNA-binding-deficient GFP-MSI1mut [30] was determined (Figure 4A). Consistent with previous studies reporting an independent RNA-binding association between MSI1 and PABP [31], the latter was co-purified with both the wild type MSI1 and the RNA-binding-deficient mutant protein (Figure 4A, upper panel). No association was observed with GFP, serving as the negative control. MSI1 was reported to associate with the NUMB mRNA [4]. In agreement, this transcript was significantly more enriched with the wild type MSI1 than with the GFP or the RNA-binding-deficient MSI1mut (Figure 4A, lower panel). In contrast to GAPDH (negative control) [32], selective association with GFP-MSI1 was also determined for the CD44 mRNA, supporting the conserved association of MSI1 and the CD44 mRNA in glioma-derived cell models.

CLIP studies in U251MG cells [12] suggested preferential 3′UTR-binding by MSI1. In agreement, the CD44-3′UTR was the most consistently “clipped” cis-element in the CD44 mRNAs in three replicates (Figure 4B). This suggested that MSI1 controls the CD44 mRNA turnover in a 3′UTR-dependent manner. Initially, this was addressed by monitoring the activity of the 3′UTR-luciferase reporters encompassing the CD44 full-length 3′UTR, expressed in KNS42 cells (Figure 4C and Figure S1A,B). Of note, alternative polyadenylation of the CD44 mRNA was reported to modulate CD44 expression. In KNS42 cells we found that this process might be underrepresented, due to high expression of the full-length 3′UTR, as determined by mRNA-seq [33]. In MSI1 knockout cells (KO), the activity of the CD44-3′UTR luciferase reporter was significantly reduced when compared to the parental cell population (Ctrl) and normalized by the activity of the reporter containing a vector-encoded (empty vector, EV) minimal 3′UTR (Figure 4C). Strikingly, the CD44-3′UTR reporter activity was significantly increased by the overexpression of GFP-MSI1 in KNS42 cells, whereas activity remained at GFP control levels when overexpressing the RNA-binding-deficient MSI1 mutant protein. These findings further suggested that MSI1 impairs the turnover of the CD44 mRNA in a 3′UTR- and RNA-binding-dependent manner. Aiming to further validate 3′UTR-dependent regulation, the vast majority of the CD44-3′UTR was deleted in the KNS42 cells using CRISPR/Cas9 and two guide RNAs, directing the nuclease to the proximal and distal ends of the CD44-3′UTR (∆CD44-3′UTR; Figure 4D). Biallelic deletion was confirmed by PCR on genomic DNA (Figure 4E).

Deletion of the 3′UTR substantially impaired the CD44 protein and mRNA expression, as revealed by Western blotting and qRT-PCR in parental (WT) and ∆CD44-3′UTR cells (Figure 4F,G). Despite an overall reduced CD44 expression, MSI1 depletion impaired CD44 protein and mRNA expression only in the parental cells, expressing the full length CD44 mRNA. Despite the overall reduced CD44 expression in ∆CD44-3′UTR cells, CD44 protein and mRNA expression remained essentially unaffected by MSI1 depletion. Collectively, this indicated that the CD44-3′UTR is central for the regulation of CD44 expression and that MSI1 promotes CD44 synthesis in a 3′UTR-dependent manner.

### 2.5. MSI1 Controls CD44 mRNA Turnover in an miRNA-Dependent Manner

**Figure 5:**
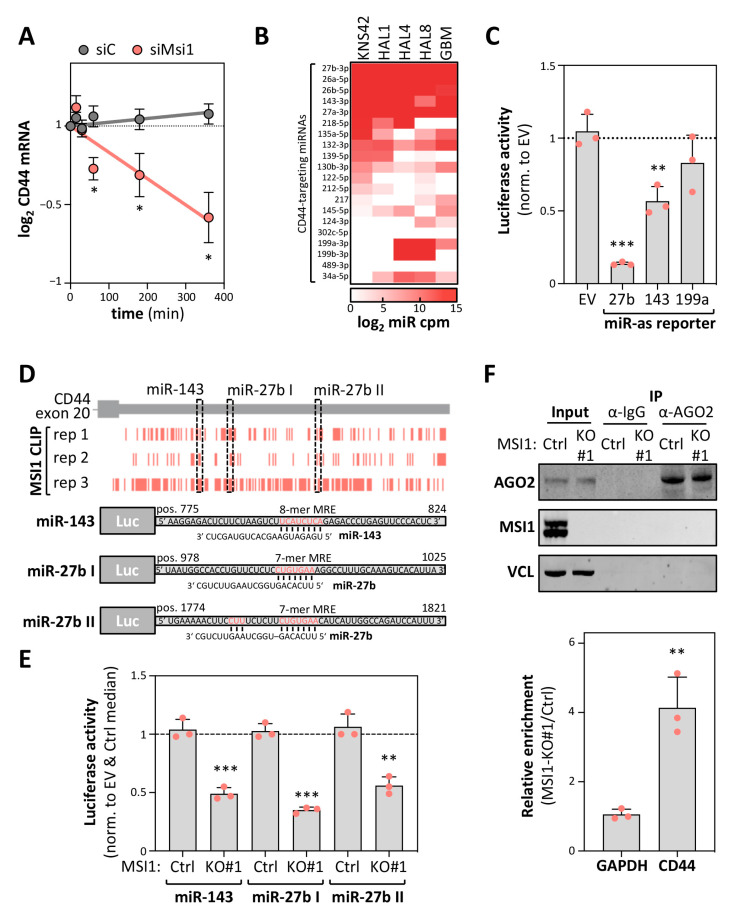
MSI1 impairs the miRNA-dependent downregulation of CD44 expression. (**A**) RT-qPCR analyses of CD44 mRNA decay in siC- or siMSI1-transfected KNS42 cells. RNA synthesis was inhibited by Actinomycin D (ActD; 5 μM) for the indicated time, 72 h post-transfection. Transcript abundance was normalized to the input levels. (**B**) Heatmap presentation of the expression of the indicated miRNAs (counts per million, cpm) in KNS42 cells, three individual isolated primary tumorspheres, HAL1, 4 and 8, and the primary GBM samples [35,36], analyzed via small RNA-seq. The 20 most abundant CD44-3′UTR-targeting miRNAs in KNS42 are indicated, predicted by TargetScan [34]. (**C**) Luciferase activity of the reporters comprising the complementary sequence to the indicated miRNAs as 3′UTR were determined in KNS42 cells. A reporter comprising the vector-encoded 3′UTR (EV) served as the control. (**D**) Peak coordinates (upper panel) derived from MSI1 CLIP [12] and mapped to the genomic region encoding for the last CD44 exon are overlaid with miRNA-recognition elements (MREs) for the indicated miRNAs. Schematic presentation of the luciferase reporters (lower panel), comprising 48 nt of the indicated regions. Respective miRNA seed regions are highlighted in red. (**E**) Luciferase activity of the indicated reporters as in (**D**) were determined in Cas9-only transfected (Ctrl) or MSI1-deleted (KO) KNS42 cell clones. (**F**) Representative Western blot analysis of the indicated proteins (upper panel) and RT-qPCR of the indicated transcripts upon AGO2 co-immunoprecipitation in Cas9-only transfected (Ctrl) or MSI1-deleted (KO) KNS42 cell clones. VCL and GAPDH served as the loading and negative controls. Error bars indicate the standard deviation from three independent experiments. Statistical significance was determined by Student’s *t*-test (***, *p* ≤ 0.001; **, *p* ≤ 0.01; *, *p* ≤ 0.05; n.s., not significant).

MSI1 was primarily reported to control mRNA translation, with only speculative evidence for a role in the regulation of transcript turnover [11,21]. Recent studies, however, indicated that MSI1 is involved in modulating AGO2/miRNA-dependent regulation of mRNA fate, at least under cellular stress [24]. In view of the 3′UTR-dependent regulation of CD44 expression, we hypothesized that MSI1 impairs miRNA-dependent downregulation of CD44 expression. This was tested by monitoring CD44 mRNA decay upon MSI1 depletion in KNS42 cells (Figure 5A). In support of a mRNA stabilizing role of MSI1, turnover of the CD44 mRNA was significantly enhanced upon MSI1 depletion in KNS42 cells treated with Actinomycin D (ActD) to block transcription. To identify the prime candidate miRNAs controlling the CD44 mRNA’s fate in glioma-derived tumor cell models and primary GBM, we analyzed the expression of miRNAs, which are predicted by TargetScan to target the CD44-3′UTR [34], by using small RNA-seq derived from cell models or publicly available data [35,36]. These analyses revealed a conserved expression of previously reported CD44-targeting miRs 27b-3p and 143-3p in primary GBM, tumorspheres as well as KNS42 cells (Figure 5B; Table S3) [37,38]. In contrast, the CD44-targeting miR-199a [39] was nearly absent in KNS42 and one tumorsphere population (HAL1). To evaluate the regulatory potency of the three aforementioned miRNAs, miRNA-antisense luciferase reporter activity was determined in KNS42 cells (Figure 5C). Reporters comprising a minimal 3′UTR (EV) without miRNA targeting sites served as the controls. Consistent with their distinct abundance, the highest repressive activity was observed for the most abundant CD44-targeting miR-27b followed by miR-143-3p, whereas miR-199a-3p reporter activity remained essentially unaffected. Intriguingly, CLIP studies suggested a partial overlap of MSI1-binding with both miR-143 as well as two miR-27b targeting sites in the CD44-3′UTR, suggesting that MSI1 impairs inhibition of CD44 expression by these miRNAs (Figure 5D, upper panel). This was tested by monitoring the activity of the luciferase reporters harboring the respective miRNA-recognition elements (MREs) with adjacent sequences (total length of 48 nucleotides) of the CD44-3′UTR (Figure 5D, lower panel), essentially as previously described [40]. In MSI1-KO KNS42 cells (KO clone #1), the activity of all these reporters was markedly reduced when compared to parental KNS42 cells and normalized to the control reporters (EV) lacking the miR-targeting sites (Figure 5E). To test the miRNA-dependent regulation of the endogenous CD44 mRNA by MSI1, we next performed an AGO2-RIP in parental and MSI1-KO cells (Figure 5F), essentially as previously described [40]. Notably, MSI1 was not co-purified with AGO2 in parental cells (Figure 5F, upper panel), contradicting the association between MSI1 and AGO2 previously proposed [24]. However, when investigating mRNA co-purification of the CD44 mRNA with AGO2 in both cell populations, we observed a substantial enrichment of the CD44 mRNA with AGO2 in MSI1-KO cells (Figure 5F, lower panel). In contrast, the GAPDH mRNA association remained essentially unchanged. In conclusion, these complementary approaches provide strong evidence that MSI1 regulates CD44 mRNA turnover in an at least partially miRNA-dependent manner.

## 3. Discussion

Several studies reported MSI1 re-expression or upregulation in various cancers [21]. According to the TCGA-provided transcriptome data, LGG (low grade glioma) and, most importantly, GBM show the highest expression of MSI1. Importantly, the MSI1 mRNA abundance remains high in recurrent GBM, suggesting a fundamental role of MSI1 in CSC maintenance and tumor recurrence [1]. Here, we present a novel mechanism of MSI1-controlled gene expression in GBM-derived cell lines—the stabilization of mRNAs by impairing miRNA-dependent downregulation. In GBM-derived cell lines as well as primary patient-derived tumorspheres, MSI1 promotes the expression of the validated GBM-CSC marker CD44 [1,26,41]. Strikingly, MSI1 is co-expressed with CD44 in single neoplastic cells at the invasive front of GBMs, supporting the view that MSI1′s role in promoting stem cell properties in gliomas essentially relies on the impairment of miRNA-directed downregulation of stem cell markers like CD44.

In line with the reported importance of MSI1 in GBM-derived cells [21], we provide evidence that MSI1 impairs a pro-neural differentiation and promotes self-renewal potential in GBM-derived cell lines and primary patient-derived tumorspheres. This supports the pivotal role of MSI1 in promoting and sustaining GBM-CSC properties, also reported in other malignancies, e.g., breast and lung cancer [6,7]. The inhibitory role of MSI1 on differentiation is furthermore observed during normal neurogenesis [42,43], and MSI1 is upregulated in neural cells when cultured under neuronal stem cell conditions [44]. Thus, MSI1 is a potent regulator of stemness in normal development as well as malignancies.

We identified a novel regulatory role of MSI1 in GBM-derived cell lines and patient-derived tumorspheres, the enhancement of stemness marker expression, here demonstrated for CD44. In addition to bulk evidence suggesting CD44 as a stem cell marker in various malignancies, including gliomas, recent scRNA-seq studies identified CD44 as a marker for a distinct lineage of glioma CSC-enriched populations [3]. Moreover, scRNA-seq indicates co-expression of MSI1 and CD44 in neoplastic cells at the invasive front of GBM [18]. In conclusion, these findings provide strong evidence that MSI1 promotes CSC properties and marker expression in neoplastic cells, in particular in GBM.

Previous studies focused on the role of MSI1 in controlling mRNA translation [4,31,45]. However, we observed that MSI1 depletion and deletion substantially impaired mRNA abundance. Intriguingly, the majority of mRNAs suggested to associate with MSI1 in CLIP studies [12] were downregulated by MSI1 depletion in GBM-derived cells. As previously reported, identified RNA-binding sites were preferentially located within 3′UTRs of the respective mRNAs, including CD44 [12]. In support of the MSI1-directed control of CD44 mRNA abundance and turnover, we demonstrate that MSI1 promotes CD44 mRNA stability, the activity of luciferase reporters harboring the CD44-3′UTR as well as abundance of the endogenous mRNA in a 3′UTR-dependent manner. This regulation is reminiscent of regulation by another oncofetal RBP, the IGF2 mRNA-binding protein 1 (IGF2BP1), which is highly expressed in aggressive malignancies [46,47]. This promotes stemness properties in cancer cells by impairing the miRNA-dependent downregulation of target mRNAs after recruiting the respective mRNAs to miRNA/RISC-devoid mRNPs [14,40,47,48]. Similar to IGF2BP1, we provide complementary evidence that MSI1 is not co-purified with AGO2 and that its downregulation or deletion enhances the miRNA-dependent downregulation of the CD44 mRNA in GBM-derived cells. Intriguingly, this regulation involves CD44 mRNA-targeting miRNAs, with reported tumor-suppressive and stemness-repressing roles like miR-143-3p and miR-27b-3p, e.g., [49,50]. Moreover, the CD44-3′UTR has frequently been described to be prone to regulation by multiple miRNAs [37,51,52]. Thus, our findings reveal an undescribed mechanism by which MSI1 promotes tumor cell stemness and highlight the pivotal role of oncofetal RBPs like IGF2BP1 and MSI1 in promoting a stem-like tumor cell phenotype by antagonizing miRNA-dependent regulation.

The conserved pro-oncogenic potential of oncofetal RBPs (oncoRBPs), such as MSI1 or IGF2BP1, suggests these as candidate targets in cancer treatment. In recent years various inhibitors interfering with RNA-binding of oncoRBPs, including MSI1 and IGF2BP1, have been described [17,53]. These lead compounds proved suitable in cellulo and in vivo to impair oncoRBP function, as demonstrated here by the inhibition of MSI1-enhanced CD44 expression and induction of tumor cell differentiation by the MSI1 inhibitor luteolin. The specificity, off-target effects, potency and efficacy of these inhibitors need to be characterized and improved. Nonetheless, the here and recently reported potency of oncoRBP-inhibitors in pre-clinical tumor models provides strong evidence that the inhibition of oncoRBPs represents promising avenues for improving cancer treatment.

Although luteolin was reported to specifically block the interaction between MSI1 and its target mRNAs, it is unlikely that only MSI1 downstream signaling is affected. In this regard, luteolin was already reported to alter antioxidant activity, modulate the MAPK and p-IGF-1R/PI3K/AKT/mTOR signaling pathways, and induce mitochondrial dysfunction in GBM, colorectal cancers and other malignancies [54,55,56,57,58]. Thus, a combination of luteolin together with already established therapies could prove to be valuable, since the current standard treatment with temozolomide and radiotherapy after tumor resection did not lead to increased survival outcome. Fortunately, recent studies already showed that luteolin could be used in combined compound application [59,60]. Interestingly, we have recently shown that MSI1 contributes to a resistance against the synergistic effect of valproic acid on temozolomide in a novel combinatorial therapeutic approach for pediatric glioma (NCT03243461) [15]. Thus, we believe that an additional application of an MSI1 inhibitor, like luteolin, would be beneficial for patients.

## 4. Materials and Methods

### 4.1. Isolation and Cultivation of Primary Tumorspheres

Fresh human GBM-derived samples were provided by the Neurosurgery Department at the University Clinics Halle, Germany. The transfer and use of samples were performed with permission of the clinical ethics committee of the University Clinics Halle (HU 1547/10-1). Primary cells were isolated and cultured as previously described [61]. In brief, under sterile conditions, resected GBM samples were placed on ice and washed with PBS to remove blood and debris. Subsequently, the samples were placed in a cell culture dish and dissected with a scalpel. Minced tissues were trypsinized in pre-warmed trypsin-EDTA for 10–15 min at 37 °C. The trypsinisation was stopped with a soybean trypsin inhibitor. The suspensions were then centrifuged at 800 rpm for 5 min. Supernatants were discarded. The remaining tissue pieces were resuspended in neural stem cell (NSC) medium (DMEM/F-12 and Neurobasal medium (1:1) supplemented with B27 supplement, bFGF (20 ng/mL) and EGF (20 ng/mL)). The cell suspensions were then filtered through 40 µm cell strainers and again centrifuged at 800 rpm for 5 min. Subsequently, the cells were cultured in NSC medium. Tumorspheres were subcultured as soon as they reached an approximate diameter of 150–200 µm. Upon trypsinisation, the cells were cultured in new NSC medium.

### 4.2. Adherent Cell Culture and Transfection

KNS42 cells (RRID: CVCL_0378) were cultured in Dulbecco’s Modified Eagle Medium (DMEM) supplemented with GlutaMAX (Thermo Fisher Scientific, Waltham, MA, USA) and 10% fetal bovine serum (FBS) at 37 °C and 5% CO_2_. Transfection of siRNAs or plasmids was performed with Lipofectamine RNAiMAX or Lipofectamine 3000 (Thermo Fisher Scientific), respectively, according to the manufacturer’s instructions. Plasmids and siRNAs are summarized in Tables S4 and S5.

### 4.3. CRISPR/Cas9 and Lentiviral Transduction

MSI1 knockout and ∆CD44-3′UTR cells were generated by using the CRISPR/Cas9-guided genome editing, as previously described [40]. For stable expression of the GFP-tagged MSI1 wildtype or a previously described RNA-binding-deficient mutant [30], pLVX plasmids (TakaraBio, Shiga, Japan) were used. Lentiviral particles were produced in HEK 293T/17 cells, as previously described [62]. Upon transduction, cells were sorted for GFP with a FACSAria II cell sorter (BD Biosciences, Franklin Lakes, NJ, USA) 48 h post-transduction. Plasmids and guide RNAs are summarized in Table S4.

### 4.4. Cell Viability, Spheroid Growth and Anoikis Resistance

If not otherwise stated, for the cell viability assay 1 × 10^4^ cells were seeded in a 96-well plate. Cell viability was determined by using the CellTiter-Glo^®^ Luminescence Cell Viability Assay (Promega, Madison, WI, USA), according to the manufacturer’s instructions. For spheroid growth, 1 × 10^4^ cells per well (24 h post-transfection) were seeded in an ultra-low attachment round bottom 96-well plate (Corning, Corning, NY, USA) using FBS-containing (10%) DMEM. Spheroid growth was monitored for 3 days with an Incucyte device (Essen BioScience, Ann Arbor, MI, USA), and viability was determined by CellTiter-Glo (Promega). For anoikis resistance, 3 × 10^4^ cells per well were seeded in an ultra-low attachment flat bottom 96-well plate (Corning, 3474) using DMEM containing 0.5% FBS. Cell viability was determined by using CellTiter-Glo (Promega) after 7 days. The half-effective concentration (EC_50_) was determined with GraphPad Prism Software (v 7.0) (San Diego, CA, USA). Formation of cell aggregates was imaged by light microscopy (Nikon TE2000-E, Tokyo, Japan).

### 4.5. Neurite Outgrowth Analysis

To analyze the neurite outgrowth, 1 × 10^4^ cells were seeded into a 96-well plate and images were taken 72 h after treatment or transfection with an Incucyte device (Essen BioScience). Neurite lengths and branch points numbers were determined by using the Incucyte NeuroTrack Scan Type tool.

### 4.6. Luciferase Assay

For the analysis of 3′end-dependent regulation, pmirGLO plasmids, comprising the full-length CD44-3′UTR, were used. A reporter containing a minimal vector-encoded 3′UTR (empty) served as the normalization control. Cells were transfected with the respective plasmid and Lipofectamine 3000 reagent (Thermo Fisher Scientific). The activities of the Firefly and Renilla luciferases were determined 48 h post-transfection by Dual-Glo (Promega). Primer sequences and plasmids for cloning are summarized in Table S4.

### 4.7. Inhibition of RNA Synthesis

For RNA decay analysis, RNA transcription was blocked by treatment with Actinomycin D (5 µM) for the indicated time points, 72 h post-transfection. RNA abundance was measured by qRT-PCR. Primer sequences for qRT-PCR are summarized in Table S6.

### 4.8. Extraction of RNA and cDNA Synthesis

Total RNA from the cell line experiments was isolated by using TRIzol. A total of 2 µg of isolated total RNA was used for the cDNA synthesis, with random hexamers and M-MLV Reverse Transcriptase (Promega), following the manufacturer’s instructions. Primer sequences for the qRT-PCR are summarized in Table S6.

### 4.9. RIP and RT-qPCR

RNA co-immunoprecipitations and RT-qPCR were performed as previously described [63]. Antibodies for the IP and primer sequences are summarized in Tables S6 and S7.

### 4.10. Western Blotting

For Western blotting, cells were lysed in 50 mM Tris–HCl (pH 7.4), 50 mM NaCl, 2 mM MgCl_2_, and 1% SDS. Protein expression was analyzed by Western blotting with the indicated antibodies (Table S7), by an infrared scanner (LICOR).

### 4.11. MSI1-CLIP Data Analysis

MSI1-CLIP peak coordinates from [12] were mapped to all annotated genes (RefSeq hg19) after lift over of genomic coordinates from UCSC GRCh18 to GRCh19. Transcripts were considered as bound by MSI1, when the respective cis-element was identified in at least two of three replicates.

### 4.12. Deep-Sequencing and Differential Gene Expression

For mRNA-seq and small RNA-seq library preparation, sequencing was performed by Novogene (Hongkong, China) or by the Deep Sequencing Facility Dresden (Technical University Dresden, Germany), respectively. For the analyses, low-quality read ends, as well as the remaining parts of the sequencing adapters were clipped off using Cutadapt (v 1.14). Reads were aligned to the human genome (UCSC GRCh38) using HiSat2 (v 2.1.0; [64]) or Bowtie2 (V 2.3.2; [65]), respectively. FeatureCounts (v 1.5.3; [66]) was used for summarizing the gene-mapped reads. Ensembl (GRCh38.89; [67]) or miRBase (v 21; [68]) was used for annotations. Differential gene expression was determined by the R package edgeR (v 3.18.1; [69]) using TMM normalization. All generated RNA-seq data were deposited at GEO (GSE159740).

### 4.13. Publicaly Available Data

The MSI1 mRNA expression data amongst all the TCGA cohorts were downloaded from cBioPortal and directly from TCGA [19,70]. CRISPR dependency scores were derived from the DepaMap database (Avana release 20Q2) [22]. ScRNA-seq data were derived from the single-cell expression atlas [28]. The evaluated miRNA-sequencing data from the GBM samples, originally generated by Skalsky et al. (2011), were derived from Hua et al. (2012) [35,36]. For the prediction of putative miRNA-binding sites within the CD44-3′UTR, TargetScan was used [34].

### 4.14. Statistics

GraphPad Prism software (v 7.0) was used for data analysis and visualization. Statistical significance was determined by Mann–Whitney tests or Student’s *t*-tests, as indicated.

## 5. Conclusions

We demonstrate for the first time that the CSC marker MSI1 is a post-transcriptional regulator of the 3′UTR- and miRNA-dependent turnover control of CD44 mRNA in GBM-derived cells of pediatric and adult origin. In these cell models, MSI1 depletion and deletion as well as luteolin treatment impair the self-renewal potential and induce pro-neural differentiation. Our findings will not only improve on the current knowledge of GBM biology and cancer stem cell markers but might contribute with a rationale for MSI1-centered therapeutic GBM targeting strategies, to improve the treatment options of this chemo/radio-resistant malignancy.

## Supplementary Materials

The following are available online at https://www.mdpi.com/2072-6694/12/12/3654/s1, Figure S1: MSI1 regulates CD44 expression in GBM-derived cells; Figure S2: CD44-3′UTR reporter construct and read coverage; Table S1: mRNA expression data (protein-coding) from primary tumorsphere lines; Table S2: Differential gene expression upon MSI1-KD in KNS42 cells; Table S3: miRNA expression data from cell lines and GBM samples; Table S4: Plasmids/guide RNAs and primers for cloning; Table S5: siRNAs; Table S6: qRT-PCR primers; Table S7: Antibodies.
