# Supplementary material for: MSI1 Promotes the Expression of the GBM Stem Cell Marker CD44 by Impairing miRNA-Dependent Degradation
Source: Cancers (Basel). 2020 Dec 5;12(12):3654. doi: 10.3390/cancers12123654 (PMC7762192; doi:10.3390/cancers12123654)
Supplement: Supplementary file 1 [file cancers-12-03654-s001.zip › _Supp_Figures_Poetschke-et-al.pdf]

Figure S1

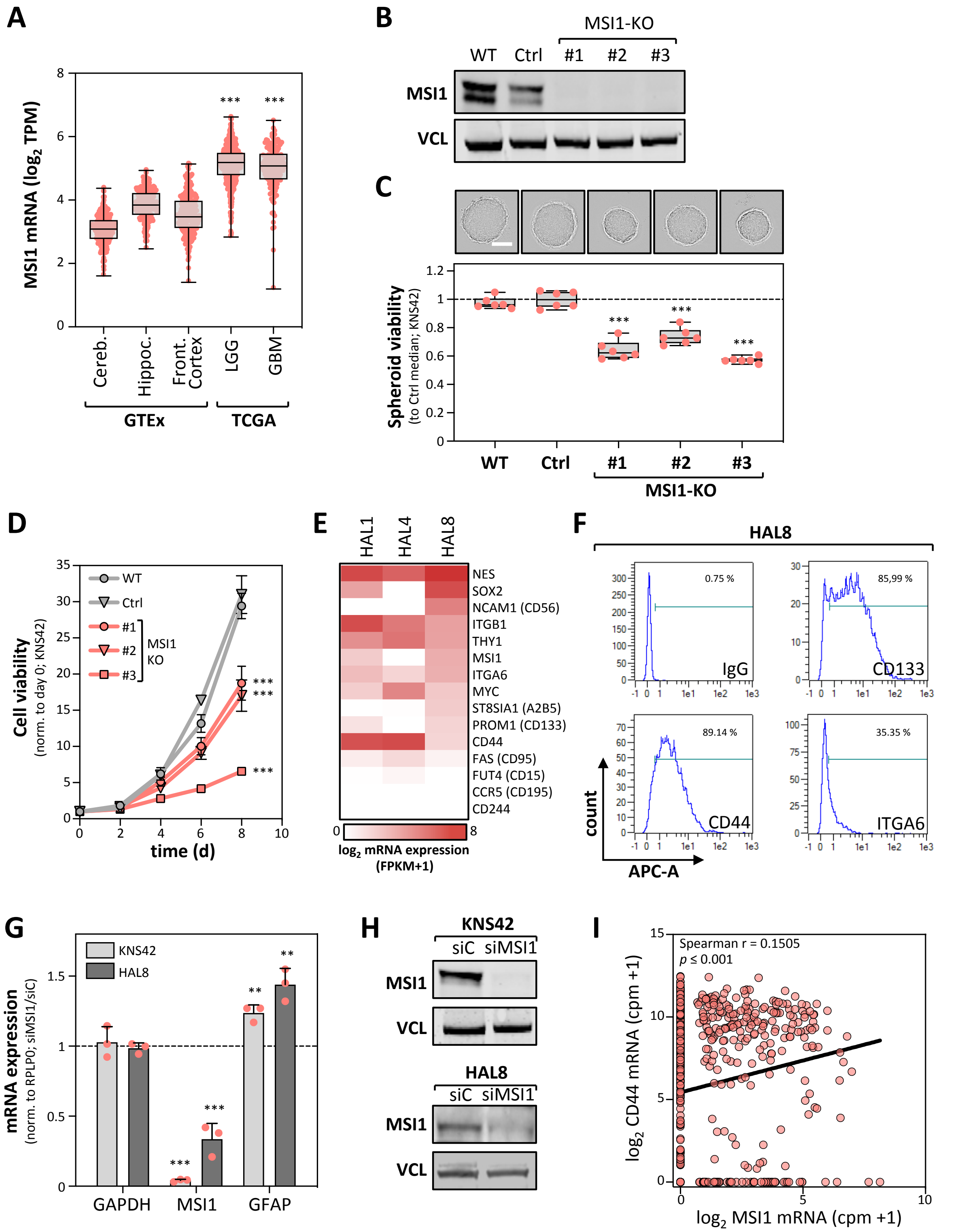

Figure S1. MSI1 regulates CD44 expression in GBM-derived cells. **A)** Box plot presentation of MSI1 mRNA expression for cerebellum, hippocampus and frontal cortex, derived from GTEx, and primary LGG and GBM, derived from TCGA. Statistical significant difference for the respective primary tumor type to each normal brain tissue was $P \leq 0.001$ (***). **B)**

Representative Western blot analysis of indicated proteins of KNS42 wildtype (WT) cells, Cas9-only transfected (Ctrl) and three individual MSI1 knockout clones. VCL served as loading control. **C)** Spheroid viability of wildtype (WT) cells, Cas9-only transfected (Ctrl) and three individual MSI1 knockout clones (n = 6 per condition), as in (B), determined by CellTiter-GLO, 72 h after seeding. Upper panel, representative images of spheroids; Scale bar, 150 µm. Lower panel: box plots show spheroid viability normalized to the median viability of Cas9-only transfected cell clone (set to one). **D)** Relative cell numbers at indicated time points for adherently growing wildtype (WT) cells, Cas9-only transfected (Ctrl) and three individual MSI1 knockout clones. **E)** Heatmap presentation depicting the mRNA expression of a panel of CSC marker genes in isolate primary tumorspheres HAL1, 4 and 8, determined by mRNA-seq. **F)** Flow cytometry analysis of expression of indicated CSC marker proteins in HAL8 cells (n = 10,000 per condition). IgG staining served as negative and background control. **G)** Relative mRNA expression determined by RT-qPCR for indicated transcripts upon transient MSI1 depletion in KNS42 cells and primary tumorspheres HAL8, 72 h post-transfection. **H)** Representative Western blot analysis of indicated proteins of KNS42 cells and primary tumorspheres HAL8 upon transient MSI1 depletion, 72 h post-transfection as in (S1G). VCL served as loading control. **I)** Correlation analysis of MSI1 and CD44 mRNA expression in single neoplastic cells (see Figure 2G, H), as determined by scRNA-seq. Spearman r, correlation coefficient; *p*, significance of correlation; trend line indicated. Error bars indicate standard deviation from at least three independent experiments. Statistical significance was determined by Mann-Whitney test (A) and Student's t-test (C, D, G) (***, P ≤ 0.001; **, P ≤ 0.01; *, P ≤ 0.05).

Figure S2

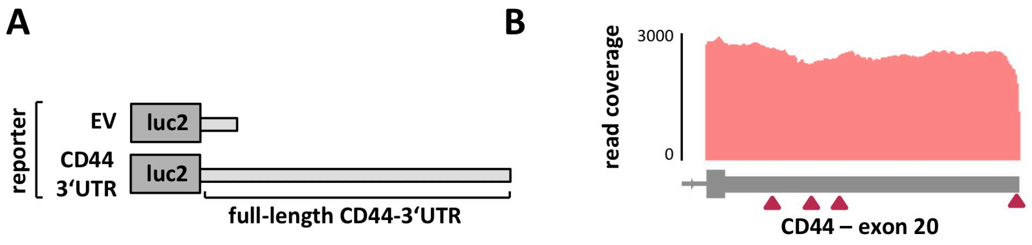

Figure S2. CD44-3’UTR reporter construct and read coverage. **A)** Schematic presenting the 3’UTRs of indicated reporters. **B)** Read coverage for the indicated genomic region derived from mRNA-seq in KNS42 cells. Red triangles indicate polyadenylation signals.
